# Role of left lateral prefrontal cortex in positive emotion regulation: Insights from dyslexia

**DOI:** 10.3758/s13415-025-01335-8

**Published:** 2025-08-15

**Authors:** Eleanor R. Palser, Nathaniel A. Morris, Christina R. Veziris, Sarah R. Holley, Amie Wallman-Jones, Ashlin R. K. Roy, Abigail E. Licata, Mieke Voges, Christa Watson Pereira, Maria Luisa Mandelli, Maria Luisa Gorno-Tempini, Virginia E. Sturm

**Affiliations:** 1 Department of Neurology, University of California San Francisco, San Francisco, CA USA; 2 Department of Psychology, Palo Alto University, Palo Alto, CA USA; 3 Psychology Department, San Francisco State University, San Francisco, CA USA; 4 Department of Psychiatry and Behavioral Sciences, University of California San Francisco, San Francisco, CA USA

**Keywords:** Adolescence, Laterality, Learning differences, Positivity, Approach, Suppression

## Abstract

Emotion regulation emerges during childhood and engages prefrontal brain systems. While most developmental studies focus on the neural underpinnings of negative emotion regulation, less is known about the neuroanatomical correlates of positive emotion regulation. In adults, prefrontal areas in the left hemisphere are critical for positive emotion regulation, but whether this association is present in children is unknown. We investigated whether smaller gray matter volume in left prefrontal regions related to worse positive emotion regulation in children with and without dyslexia. Because dyslexia is a neurodevelopmental disorder of reading in which there may be greater variability in left prefrontal structures, it offers a unique window into the neural basis of positive emotion regulation. Sixty-nine children (ages 7–13) were asked to hide their feelings while watching film clips that elicited either amusement or disgust, and evaluate their emotion regulation performance. Parents reported on children’s everyday emotion regulation. Across the sample, children with smaller volumes in left prefrontal regions (i.e., lateral orbitofrontal cortex and dorsolateral prefrontal cortex) had greater facial behavior (i.e., suggesting worse positive emotion regulation) yet more positive self-reported emotion regulation success during the amusement trial (i.e., suggesting more favorable self-evaluation). These regions did not relate to disgust regulation, however. Children with more positive views of their emotion regulation success also had better everyday parent-reported emotion regulation. Results suggest that positive emotion regulation in childhood relies on left prefrontal regions and a more optimistic view of one’s emotion regulation abilities, regardless of one’s actual emotional behavior, may confer real-world advantages.

**Supplementary Information:**

The online version contains supplementary material available at 10.3758/s13415-025-01335-8.

## Introduction

Positive emotions are brief states that play important roles in everyday life (Shiota et al., 2017). While negative emotions encourage withdrawal from harmful stimuli and safety-seeking behavior, positive emotions encourage social engagement, reward-seeking, and exploration (Fredrickson, 2013; Fredrickson & Levenson, 1998; Shiota et al., 2011). To be adaptive, however, positive emotions must not be unbridled. Emotion regulation refers to the processes by which people select and modify which emotions they have and when (Gross, 2015). Prior studies have often focused on negative emotion regulation, in part because of its role in clinical conditions including anxiety and depression (Kring & Sloan, 2009), but positive emotion regulation is also critical for decision-making and goal attainment. When mild, difficulties with positive emotion regulation can increase distractibility (Dreisbach & Goschke, 2004) and impulsivity (Cyders et al., 2007; Sperry et al., 2016), and impede skillful social interaction (Erber et al., 1996; Kalokerinos et al., 2014). In contrast, more significant difficulties with positive emotion regulation may lead to problems with interpersonal boundaries, risk-taking, and mania (Gruber et al., 2011).

The regulation of positive emotions, like negative emotions, is complex. As multicomponential processes, emotions are accompanied by changes in experience, facial behavior, and physiological responding (Ekman, 1992; Izard, 1977) that are loosely coupled in both adults (Levenson et al., 1990; Mauss et al., 2005) and children (Casey, 1993; Eisenberg et al., 1988; Hubbard et al., 2004; Underwood & Bjornstad, 2001). Emotion regulation processes can modify the activity in one or more of these domains (Gross, 2002), but even successful regulation of one domain can have distinct effects in the other domains. While reappraisal, which involves changing one’s thinking about a situation, can reduce all types of emotional experience, suppression, which involves inhibiting expressive behavior, has more complicated effects (Gross & John, 2003; Katsumi & Dolcos, 2020). Some studies (Bush et al., 1989; Dan-Glauser & Gross, 2011; Gross & Levenson, 1997; McCanne & Anderson, 1987; Stepper & Strack, 1993; Strack et al., 1988; Vrtička et al., 2011), but not all (Kalokerinos et al., 2015; Korb et al., 2012), find that suppression reduces positive emotional experience more than negative emotional experience. Similarly, people who are dispositionally less expressive tend to experience less positive emotion (Gross & John, 2003).

Variability in positive emotion regulation across people may reflect structural differences in the prefrontal cortex (PFC), a brain region that plays a central role in affective, cognitive, and behavioral control (Aron, 2007; Aron et al., 2014; Bush et al., 2000; McRae et al., 2012; Ochsner & Gross, 2005; Wager et al., 2008). The lateral PFC is especially important for inhibitory processes (Aron & Poldrack, 2006; Kringelbach & Rolls, 2004; Öngür & Price,^2000^; Tabibnia et al., 2011), and functional neuroimaging studies typically find that activation of lateral PFC regions reduces activity in subcortical structures important for emotion generation, such as the amygdala (Berkman et al., 2009; Lieberman et al., 2007; McRae et al., 2010). Within the lateral PFC, ventral regions, including the lateral orbitofrontal cortex (OFC), modulate our emotions, thoughts, and actions (Aron et al., 2003; Chambers et al., 2006; Cunningham & Zelazo, 2007; Koenigsberg et al., 2010; Lieberman et al., 2007; Ochsner et al., 2004; Petrides et al., 2005). The dorsolateral PFC, with connections to lateral parietal cortex, also contributes to emotion regulation (Golkar et al., 2012; Ochsner et al., 2009; Ochsner & Gross, 2005) by promoting behavioral and mental flexibility (Champod & Petrides, 2007; MacDonald et al., 2000). As the PFC continues to develop in the early years of life, children become increasingly adept at emotion regulation. Effortful inhibitory control, which is important for the suppression of behavioral and emotional responses (Carlson & Wang, 2007; Kochanska et al., 2000; Moriguchi & Hiraki, 2013), emerges between ages 2 and 5 years and continues to mature throughout childhood (Eisenberg et al., 2010). Even at this early age, children can control their emotional expressions and are aware of these efforts (Cole et al., 2009; Davis et al., 2010). As children enter adolescence, strengthened structural connections between the PFC and subcortical regions further enhance suppression abilities (Ahmed et al., 2015; Vilgis et al., 2018).

Although much remains unknown, positive emotion regulation may rely predominantly on PFC structures in the left hemisphere. Whereas the right hemisphere is critical for negative emotions and avoidance behaviors (Grimm et al., 2008; Vergallito et al., 2018), left-hemisphere systems are important for positive emotions and approach behaviors (Ahern & Schwartz, 1985; Davidson et al., 2000; Davidson & Fox, 1982; Herrington et al., 2005; Murphy et al., 2003; Sackeim et al., 1982; Shdo et al., 2022; Sturm et al., 2015). In our prior research, we found that older adults who have smaller gray matter volume in left-lateral OFC (as well as other left-sided structures, such as the left dorsal anterior insula and left superior temporal gyrus) had elevated positive emotional behavior and experience while viewing amusing videos and photographs (Shdo et al., 2022; Sturm et al., 2015). These studies, although conducted in people in the later decades of life, suggest that variability in left PFC volume relates to the intensity of positive emotional behavior and experience. As inhibitory networks anchored by the lateral PFC continue to mature throughout childhood, adolescence, and early adulthood (Casey et al., 2005; Gogtay et al., 2004; Vijayakumar et al., 2014), emotion regulation improves (McRae et al., 2012; Silvers et al., 2015). Most of the previous research on suppression in children and adolescents has focused on negative emotions (see Gross and Cassidy, 2019, for a review), with very little research on positive emotion suppression.

Dyslexia is a neurodevelopmental condition that predominantly affect the left hemisphere (Galaburda, 1994; Hoeft et al., 2007; Vanderauwera et al., 2017; Vandermosten et al., 2012), providing a unique opportunity to uncover the neural correlates of positive emotion regulation, which may be difficult to discern in typically developing samples due to limited behavioral and anatomical variability. Dyslexia is primarily a disorder of reading (Shaywitz, 1998), but many individuals with dyslexia also have trouble with impulsivity (Duranović et al., 2023) and distractibility (Facoetti & Molteni, 2001; Smith-Spark et al., 2004), behavioral challenges that may relate to smaller or weaker lateral PFC systems. Affective symptoms, which too are common in dyslexia, may also reflect problems with emotion regulation (Boyes et al., 2020; Carroll et al., 2005; Rodriguez & Routh, 1989) and underlying lateral PFC structures. In our prior work, we found that children with dyslexia had greater emotional reactivity while watching both negative and positive emotional film clips (Sturm et al., 2021), but whether their heightened emotional reactions were due to poor emotion regulation or enhanced emotional reactivity was difficult to disentangle.

We investigated whether variability in gray matter volume in lateral PFC regions related to positive emotion regulation abilities in children with and without dyslexia. While most studies measure emotion regulation with self- or parent-report measures (e.g., Silvers et al., 2012), we used a laboratory-based task (e.g., Gross & Levenson, 1993; 1997; Gyurak et al., 2012; Shiota & Levenson, 2009) to quantify positive emotion regulation in children with and without dyslexia. Participants were instructed to hide their reactions while they watched a film clip that evoked amusement. A reactivity task in which they just watched and were not instructed to hide their reactions served as a “no regulation” control condition. They were also asked to hide their reactions while they watched a film clip that elicited disgust, and this trial served as a negative emotion regulation comparison condition. Facial behavior was used as an objective index of emotion regulation (where lower facial behavior reflected better emotion regulation), and self-report measures were used as subjective measures of emotion regulation success. To examine whether laboratory-based measures of positive emotion regulation abilities were associated with real-world behavior, parents reported on their child’s emotion regulation in everyday life.

## Methods

### Participants

Sixty-nine participants (45 children with dyslexia and 24 typically developing children) recruited from the University of California, San Francisco (UCSF) Dyslexia Center were included in the present study. All were fluent English speakers between the ages of 7 and 13 years. The study protocol was approved by the UCSF Human Research Protection Program. Participants provided verbal assent; guardians provided written informed consent. Procedures were conducted in accordance with the 1964 Declaration of Helsinki.

Children with a history of dyslexia underwent a comprehensive multidisciplinary evaluation, including a clinical interview and standardized neuropsychological and academic testing. Participants in the dyslexia group had prior and confirmed diagnoses at the time of testing. Most of the children with dyslexia (64%) attended specialist schools. Attention deficit hyperactivity disorder (ADHD) was common in the dyslexia group (33%), reflecting the high co-occurrence of these conditions, with prior studies suggesting that 25–40% of children with one disorder meet criteria for the other (August & Garfinkel, 1990; Semrud-Clikeman et al., 1992; Willcutt & Pennington, 2000). The typically developing children, who reported no history of symptoms associated with neurodevelopmental disorders, including ADHD, attended local mainstream schools, and completed an abbreviated evaluation that included abridged neuropsychological and academic testing. Children were excluded if they had a history of acquired brain injury, known genetic condition that impacts cognition, psychiatric disorder, or neurodevelopmental condition (other than dyslexia or ADHD in the dyslexia group).

### Neuropsychological and reading assessment

All participants completed Matrix Reasoning, a test of nonverbal reasoning from the Wechsler Abbreviated Scale of Intelligence (WASI; Wechsler, 1999) and performed at least above the ninth percentile (i.e., above the impaired range). Single-word reading was assessed with the timed Test of One-Word Reading Efficiency – Second Edition, which provides timed measures of sight word efficiency and phonemic decoding efficiency (TOWRE-2; Torgesen et al., 2012).

The children with dyslexia completed additional tests of language and reading. Single-word reading was assessed with untimed Letter-Word Identification and Word Attack measures from the Woodcock-Johnson IV (Schrank et al., 2014). Paragraph reading was assessed using the Gray Oral Reading Test – Fifth Edition (GORT-5; Wiederholt & Bryant, 2012). Testing confirmed that all children with dyslexia had at least one low reading score (≤25^th^ percentile), and most of the children with dyslexia (80%) fell below the tenth percentile on at least one reading measure. Five children with dyslexia did not complete all academic testing due to time constraints (one had missing TOWRE-2 and GORT-5, two had missing GORT-5, two had missing Woodcock-Johnson IV); however, all five displayed at least one low reading score (≤25^th^ percentile) on completed measures and, thus, were retained in the sample.

### Laboratory assessment of emotion regulation

#### Procedure

We used a film-based approach to emotion induction previously used in studies of children with neurodevelopmental disorders (Sturm et al., 2021). Participants were seated in a comfortable chair in a well-lit testing room. All instructions were presented visually and orally. Visual stimuli were presented on a 21.5-inch computer monitor placed 4.25-feet in front of them. All audiovisual instructions were presented using E-Prime (version 3.0, Psychology Software Tools, Pittsburgh, PA). During the assessment, the experimenter left the testing room, observing the participant from a nearby control room with a semiconcealed camera and communicating via an intercom system. Participants were informed they would be videorecorded. They completed a battery of tasks designed to assess emotional reactivity, empathy, and emotion regulation. All participants completed the tasks in the same order. Only the emotion regulation task and a portion of the emotional reactivity task were analyzed here.

#### Tasks

##### Emotion word knowledge task

At the beginning of the laboratory assessment, participants completed a task that assessed whether they understood the meaning of each of the emotion terms that would be used throughout the assessment. Participants were asked, “For each question, you will see an emotion word at the top of the screen. Pick the situation where you would feel the emotion.” Participants were presented with three choices for each emotion term. The experimenter reviewed any questions that participants answered incorrectly and explained the correct responses to them at the end of the task. This step was taken to ensure that participants understood all emotion terms that would be used throughout the emotion regulation task. If participants asked for clarification about the meaning of any word later in the session, the experimenter reminded them of the meaning as often as needed.

##### Emotion regulation task

At the beginning of the emotion regulation task, participants were presented with the following instructions, “In the next task, you will watch movies. Hide your reaction so that no one would know how you feel when you watch the movie. Before each movie, you will see an ‘X’ on the screen. Please relax and try to clear your mind when you see the ‘X’ on the screen. Watch the ‘X’ please.”

Each trial began with a 30-second pretrial baseline period in which participants viewed a black “X” on a white screen. They then watched a 90-second film clip that evoked a specific positive (i.e., amusement) or negative (i.e., disgust) emotion. We elicited amusement and disgust, because these emotions are often accompanied by observable, distinct changes in facial behavior and can be difficult to regulate (Gyurak et al., 2012). The amusement clip showed two babies laughing and smiling while being sprayed with water (*YouTube* clip). The disgust clip showed a woman using her mouth to search through a plate of worms (*Fear Facto*r; Series 2, Episode 17). Pilot testing in an independent sample of children indicated that the film clips elicited the target emotions. Each participant viewed the disgust clip then the amusement clip. Each trial ended with a 30-second posttrial baseline period in which participants again viewed a black “X” on a white screen.

After viewing each clip, participants were asked a series of questions. First, they responded to a multiple-choice question about the content of the clip to ensure that they had paid attention during the task. Participants were asked what happened in the clip and given three response options to choose from. Response options for the amusement trial were a) Children laughed and smiled, b) Telephones rang, and c) Children threw trash. Response options for the disgust trial were a) A horse ran away, b) People ate worms, and c) People broke a window. Second, they rated their experience of 11 distinct emotions during the clip on a three-point scale using the response options, “*Not at all*,” “*A little*,” or “*A lot*.” We focused on self-reported amusement and disgust in analyses, but all 11 types of emotional experience are reported in the supplementary materials. Third, participants rated their emotion regulation success by indicating how well they had hidden their reaction to the clip on a five-point scale using the response options, “*Very bad*,” “*Bad*,” “*OK*,” “*Good*,” or “*Very good*,” providing a measure of self-reported emotion regulation success. Finally, participants were asked on a three-point scale if they had seen the clip before, using the response options, “*Not seen before*,” “*Not sure*,” or “*Seen before*,” providing a measure of their prior familiarity with the stimulus. Participants provided verbal responses, which were recorded by the experimenter.

##### Emotional reactivity task

Prior to the emotion regulation task, emotional reactivity was assessed in most participants (78%, *n* = 30 children with dyslexia, *n* = 23 typically developing children) with a film-viewing task in which they watched five film clips that each elicited a specific emotion. Each trial of the emotional reactivity task began with a 60-second resting baseline period in which participants watched a black “X” on a white computer screen. They then viewed an approximately 90-second film clip that elicited a specific positive (i.e., amusement, awe, love) or negative (i.e., disgust, sadness) emotion. For example, the amusing film clip showed a baby laughing while watching someone ripping up paper while the disgust film clip showed inside a patient’s ear being cleaned. These film clips have been shown to reliably elicit target emotions in children and adolescents (Sturm et al., 2021). At the beginning of the task, participants were instructed, “Now you will watch some movies. After each movie, we will ask you some questions. We want to know how YOU feel while watching the movie. If you find the videos too upsetting, please close your eyes. Before each movie, you will see an ‘X’ on the screen. Please relax and try to clear your mind when you see an ‘X’ on the screen. Let's begin. Watch the ‘X,’ please.” This task was included to account for participants’ emotional reactivity for the neuroimaging analyses.

#### Measures

##### Facial behavior

Videotaped recordings of the emotional reactivity and emotion regulation tasks were coded with Noldus version 13.0 software (Noldus Technologies, Leesburg, VA). In line with previous studies (Palser et al. 2021, 2024; Sturm et al., 2021; Wallman-Jones, in press), participants’ facial behavior during the first 30 seconds of each clip was coded on a second-by-second basis using a modified version of the Emotional Expressive Behavior coding scale (Gross & Levenson, 1995). We selected this 30-second “hotspot” to capture the period of the clips when expressive behavior was greatest, based on piloting, and to ensure a comparable window between the reactivity and regulation tasks. The original system was developed to capture a broad range of facial behaviors, with a particular focus on emotional behaviors. The modified scale used here combines the categorical aspects of the Emotional Expressive Behavior scale and the Facial Affect Coding System (Ekman & Friesen, 1976). To enable greater coding precision, each emotion category was defined by specific facial movements as determined by prior research (Campos et al., 2013; Keltner et al., 2019; Matsumoto et al., 2008). Coders rated the presence of the following categories: interest, concentration, anger, sadness, disgust, fear, contempt, happiness/amusement, surprise, and embarrassment, relative to each participant’s resting neutral facial expression. All behaviors were rated on a three-point intensity scale: 1 (*slight but noticeable*), 2 (*moderate*), or 3 (*strong*). When none of these behaviors were present, “no emotion” was recorded. Codes were mutually exclusive; therefore, blends of emotion were not permitted.

Thirty percent of the videos were rated by multiple coders; interrater reliability was excellent (Cohen’s kappa =.76; Cohen, 1960; Fleiss et al., 1981). We computed a total facial behavior score for each trial by summing the intensity scores of all codes across the 30 seconds. Less facial behavior, as reflected by lower total facial behavior scores, indicated better emotion regulation.

##### Self-report questions

A total emotion word knowledge score was calculated for each participant by summing their total correct responses; higher scores indicated greater knowledge of emotion terms (maximum score = 15). The attention check for the emotion regulation task was scored as 1 (correct) or 0 (incorrect). Each type of self-reported emotional experience was coded as 0 (not at all), 1 (a little), or 2 (a lot). Each participant’s self-reported evaluation of their emotion regulation success was coded as 0 (very bad), 1 (bad), 2 (OK), 3 (good), and 4 (very good). Responses to the familiarity question were coded as 1 (yes), 0 (no), and 2 (not sure).

### MRI data acquisition and preprocessing

Participants were scanned within 90 days of the emotion regulation task. Structural MRI scans were acquired at a field strength of 3 Tesla with a T1-weighted magnetization-prepared rapid gradient-echo (MPRAGE) sequence (160 sagittal slices; slice thickness = 1.0 mm; field-of-view [FOV] = 256 x 240 mm^2^; matrix size = 256 x 240; voxel size = 1.0 x 1.0 x 1.0 mm^3^; repetition time [TR] = 2300 ms; echo-time [TE] = 2.98 ms; flip angle = 9°) using either a TIM Trio scanner equipped with a 12-channel head coil (*n* = 28, 49% of children) or a Prisma scanner equipped with a 64-channel head coil (*n* = 29, 51% of children) (Siemens, Iselin, NJ). Head movements were minimized by stabilizing the participant’s head with cushions.

We preprocessed T1-weighted images using FreeSurfer (version 6.0; http://surfer.nmr.mgh.harvard.edu/) in accordance with a standard auto-reconstruction algorithm, which involved nonuniform intensity normalization, removal of non-brain tissue, affine registration to Montreal Neurological Institute (MNI) space and Talairach transformation, and segmentation of gray and white matter tissue (Dale et al., 1999; Fischl et al.,1999a; 1999b). Segmentation results were visually inspected. Cortical parcellation and volume measurement were performed by using the Desikan-Kyliany Atlas, which comprises 34 cortical regions of interest (ROIs) per hemisphere (Desikan et al., 2006). Given the central role of the PFC in emotion regulation (Kalisch, 2009; McRae et al., 2012; Ochsner et al., 2012), we focused on gray matter volume in nine PFC regions of interest: caudal middle frontal gyrus, rostral middle frontal gyrus, caudal anterior cingulate cortex, rostral anterior cingulate cortex, lateral orbitofrontal cortex, medial orbitofrontal cortex, pars opercularis, pars triangularis, and pars orbitalis in the left and right hemispheres. Variability in volumes between the scanners was removed using the ComBat harmonization method (Fortin et al., 2018; Johnson et al., 2007).

### Parent-reported everyday emotion regulation

Parents completed the Behavior Assessment System for Children – Second Edition (BASC-2) parent rating scales (Reynolds & Kamphaus, 2004). The BASC-2 is a standardized, well-validated, multidimensional rating system that assesses skills and personality traits as well as adaptive and problem behaviors. The child form (ages 6–11) consists of 160 items; the adolescent form (ages 12–21) consists of 150 items. The BASC-2 scoring algorithm standardizes participants’ scores within their age group, making scores on both forms equivalent. Parents rated each item according to the frequency of the behavior on a four-point scale, ranging from N (*never*), through S (*sometimes*), O (*often*), to A (*almost always*). Item raw scores were summed to obtain subscale scores for 14 behavioral domains. We focused on the “Emotional Self-control” subscale, which captures behaviors related to challenges with regulating emotion. Example items include: “loses temper too easily,” “acts out of control,” “changes moods quickly,” and “is easily upset.” Each participant’s subscale score was converted into a standardized *T* score (mean [*M*] = 50; standard deviation [*SD*] = 10). High scores represent worse emotion regulation; with *T* scores between 60 and 69 considered at-risk, and scores ≥70 considered clinically significant. Scores on this measure were available for 38 participants (30 dyslexia and eight typically developing); parents of the remaining participants declined to complete this measure.

### Data analysis

Data analyses were conducted in R Studio (R version 4.2.0; R Core Team, 2022) and SPSS (version 29.0.0.0). Two-tailed tests were used throughout.

#### Sample characteristics

We used Student’s *t*-tests to compare the two groups (dyslexia and typically developing) on age, nonverbal reasoning, emotion word knowledge, and reading measures that were available across the sample. We used a chi-squared test to examine group differences in sex.

#### Emotion regulation task

All participants provided correct responses to the attention check, suggesting they attended during the task. None of the participants reported seeing the amusement clip before, and familiarity with the disgust clip was also low (two participants with dyslexia and one typically developing participant reported seeing it prior). Thus, these questions were not analyzed further. Mann-Whitney-Wilcoxon tests were used to examine group differences in self-reported experience of amusement and disgust. Because these data only used a restricted three-point response scale, thus limiting their suitability for linear analyses, we did not analyze associations with regional brain volumes.

To assess the validity of participants’ self-reported emotion regulation, we used bivariate Pearson correlations (or their nonparametric equivalent) to examine the associations between total facial behavior and self-reported emotion regulation success in each trial. In these correlational analyses, a negative association would indicate that participants with lower total facial behavior had greater self-reported emotion regulation. In follow-up bivariate correlation analyses, we also examined whether there were associations between the various self-report measures (i.e., experience, emotion word knowledge, and emotion regulation success for the amusement and disgust regulation trials). Lastly, we performed an analysis of variance (AVOVA; controlling for age, sex, and nonverbal reasoning) to determine whether the groups differed on behavioral and self-reported emotion regulation success during the amusement and disgust trials.

#### Neural correlates of positive emotion regulation

First, we used ANOVA to test for group differences in all ROIs (controlling for total intracranial volume [TIV; to control for head size], age, sex [dummy-coded for two sexes], and nonverbal reasoning). Second, as in previous studies (Sturm et al., 2013), we used forward entry linear regressions to investigate the neural correlates of total facial behavior and self-reported emotion regulation success during the amusement trial. This approach was chosen to reduce the number of comparisons, which controls the false discovery rate and reduces model overfitting. Forward entry models start with an empty model and iteratively add predictor variables that improve model fit at each step until no further improvement is observed. Predictor variable entry is determined based on accounting for significant variance above and beyond the control variables. This ensured that the brain regions with greatest involvement in positive emotion regulation were identified. We entered TIV, nonverbal reasoning, group (dummy-coded for two diagnostic groups), age, and sex in step one. In step two, the ROIs competed for entry into the model and entered if they predicted the emotion regulation variable of interest (total facial behavior or self-reported emotion regulation success) at *p* ≤.05. Variables were removed by the model at *p* ≥.100. For the analysis of total facial behavior, we entered the additional variable of total facial behavior during the amusement trial of the emotional reactivity task (i.e., the “just watch” condition) in step one. Controlling for emotional reactivity ensured that the identified regions predicted emotion regulation above and beyond individual differences in positive emotional reactivity. To reduce collinearity effects between homologous structures, we conducted separate analyses for each hemisphere (Sturm et al., 2013). Third, we used simple linear multiple regressions, controlling for TIV, nonverbal reasoning, group, age, sex, and disgust reactivity to confirm that gray matter volume in regions that predicted emotion regulation during the amusement trial did not also predict emotion regulation during the disgust trial.

#### Parent-reported everyday emotion regulation

We performed multiple linear regression analyses to determine whether lower total facial behavior or greater self-reported emotion regulation success during the amusement trial predicted real-world emotion regulation, per parent-report. These analyses were conducted across the sample and controlled for group, age, sex, and nonverbal reasoning.

## Results

### Sample characteristics

The sample comprised typically developing children and children with dyslexia, of whom 37 were boys and 31 were girls, with a mean age of 10.4 (*SD* = 1.7). Their nonverbal reasoning ranged from low average to very superior (percentile *M* = 72.1, *SD* = 21.3). The household annual income of the sample ranged from $60,000 to ≥$500,000, with a median income bracket of $250,000 to 299,999, suggesting a relatively high socioeconomic status across the sample. Medication usage was minimal, with only two participants (both with dyslexia) taking allergy medications, and six participants (all with dyslexia) taking stimulants at the time of the study. None reported taking anxiolytics, antidepressants, or beta-blockers. There were no differences between the groups in age, sex, or nonverbal reasoning. As expected, children with dyslexia had poorer reading scores than typically developing children as measured by both sight word efficiency and phonemic decoding efficiency (Table 1). In general, participants’ knowledge of emotion words was good, with a mean accuracy of 13 of 15 words answered correctly across the sample, despite the children with dyslexia having poorer emotion word knowledge than the typically developing children (Table 2).

**Table 1:** Demographic and academic characteristics of the sample

Measure	Dyslexia			Typically developing			
	Range	*M*	*SD*	Range	*M*	*SD*	*Statistic*
*N*	45			24			--
Sex (M:F)	25:20			13:11			χ^2^(1) = 2.18, *p* =.140
Race^a^	64% white, 4% Asian/Pacific Islander, 9% Multiracial, 22% NA			40% white, 7% Asian/Pacific Islander, 13% Multiracial, 40% NA			--
Age^b^	7-13	10.3	1.8	7-13	10.5	1.7	*t*(66) = 0.02, *p* =.986
Nonverbal reasoning ability^c^	16-99	72.3	21.6	16-98	69.5	23.6	*t*(66) = 1.28, *p* =.204
Letter-word identification^d^	0.1-79	31.0	25.0	--	--	--	--
Word attack^d^	1-95	35.1	25.3	--	--	--	--
Sight word efficiency^e^	0.1-75	17.2	20.6	16-98	56.5	27.2	*t*(66) = −4.00, *p* <.001
Phonemic decoding efficiency^e^	0.4-45	13.6	13.0	18-98	56	23.5	*t*(66) = −6.02, *p* <.001
Reading rate^f^	0.4-75	19.0	17.4	--	--	--	--
Reading accuracy^f^	1-50	10.3	9.9	--	--	--	--
Reading fluency^f^	0.4-50	12.8	11.4	--	--	--	--
Reading comprehension^f^	1-63	20.7	17.1	--	--	--	--

Typically developing children completed an abridged reading evaluation. ^a^NA = not available, where parents declined to report child’s race. ^b^Age reflects chronological age, given in years. ^c^Nonverbal reasoning ability reflects percentile score on the Wechsler Abbreviated Scale of Intelligence, Matrix Reasoning subtest (WASI; Wechsler, 1999﻿). ^d^Letter-word identification and word attack reflect percentile scores on the Woodcock-Johnson IV (Schrank et al., 2014) subscales of the same names. ^e^Sight word efficiency and phonemic decoding efficiency reflect percentile scores on the Test of One-Word Reading Efficiency – Second Edition (TOWRE-2; Torgesen et al., 2012) subscales of the same names. ^f^Reading rate, accuracy, fluency, and comprehension scores reflect percentile scores on the Gray Oral Reading Test – Fifth Edition (GORT-5; Wiederholt & Bryant, 2012)

*M* = mean; *SD* = standard deviation

**Table 2:** Group averages for emotion word knowledge, parent-reported emotion dysregulation during everyday life, and laboratory-based emotion regulation measures during the amusement and disgust trials

Variable	Dyslexia	Typically developing	Statistic
Emotion word knowledge	13.0 (1.1)	13.9 (1.2)	*F*(1,64) = 10.55, *p* =.002*
Parent-reported emotion regulation	49.2 (29.4)	37.1 (24.0)	*F*(1,34) = 0.46, *p* =.500
**Amusement trial**			
Total facial behavior	1.7 (1.3)	1.4 (1.4)	*F*(1,63) = 0.12, *p* =.729
Self-reported emotion regulation success	2.3 (1.2)	2.4 (1.0)	*F*(1,63) = 0.09, *p* =.917
**Disgust trial**			
Total facial behavior	1.5 (1.6)	0.9 (1.3)	*F*(1,61) = 3.08, *p* =.085
Self-reported emotion regulation success	1.9 (1.0)	2.5 (1.0)	*F*(1,61) = 6.11, *p* =.016*

Means and standard deviations (in parentheses) are presented. Statistical analyses of group differences were performed using analysis of variance with group, age, sex, and nonverbal reasoning entered as factors. Parent-reported emotion regulation reflects percentile score on the Behavioral Assessment System for Children – Second Edition (BASC-2; Reynolds & Kamphaus, 2004) emotional self-control subscale; higher scores indicate more challenges with emotion regulation. Asterisk indicates group difference at *p* <.05

### Emotion regulation task

#### Behavior and experience during the positive emotion regulation trial

Across the sample, participants displayed moderate to high levels of amusement, interest, and embarrassment facial behavior during the amusement trial (Supplementary Table 1), but the groups did not differ in their total facial behavior, *F*(1,63) = 0.12, *p* =.729 (Table 2). Participants in both groups also reported experiencing similar levels of intense amusement during the trial, *W* = 539.5, *p* =.100, but low amounts of other positive emotions, including excitement, love, surprise, and awe (Supplementary Table 3). During the amusement trial, the groups reported a wide range of emotion regulation success scores (Supplementary Table 2), but the mean levels were similar, *F*(1,63) = 0.09, *p* =.917 (Table 2), with most reporting “OK” levels of emotion regulation success. Lower total facial behavior (i.e., greater emotion regulation) during the amusement trial correlated with greater self-reported emotion regulation success, *r*_*s*_(66) = −0.34, *p* =.005 (Supplementary Fig. 1a), suggesting alignment between behavioral and subjective markers of positive emotion regulation. Follow-up analyses indicated that there were no associations between self-report measures of emotional experience, facial behavior, and emotion regulation success during the amusement trial (Supplementary Table 5), but caution is advised in interpreting these results given the limited range of the self-reported emotional experience scales. Emotion word knowledge was not associated with self-reported emotional experience or emotion regulation success during the amusement trial (Supplementary Table 5).

#### Behavior and experience during the negative emotion regulation trial

For the disgust trial, total facial behavior, *F*(1,61) = 3.08, *p* =.085, and disgust experience was comparable between the groups, *W* = 536.5, *p* =.963. Overall, participants displayed moderate levels of disgust and concentration but produced less total facial behavior than during the amusement trial (Supplementary Table 1). They reported moderate levels of disgust experience, as well as low levels of surprise. Participants reported a range of emotion regulation success (Supplementary Table 2), but those with dyslexia reported lower emotion regulation success during the disgust trial than typically developing participants, *F*(1,61) = 6.11, *p* =.016. Lower total facial behavior (i.e., greater emotion regulation) across the sample during the disgust trial correlated with greater self-reported emotion regulation, *r*_*s*_(64) = −0.36, *p* =.003 (Supplementary Fig. 1b). Greater self-reported emotion regulation during the disgust trial was also associated with less disgust experience (Supplementary Table 4). There were no associations between disgust experience and either total facial behavior or disgust facial behavior during this trial. Emotion word knowledge was not associated with self-reported emotional experience or emotion regulation success during the disgust trial (Supplementary Table 5).

### Neural correlates of positive emotion regulation

**Fig. 1:**
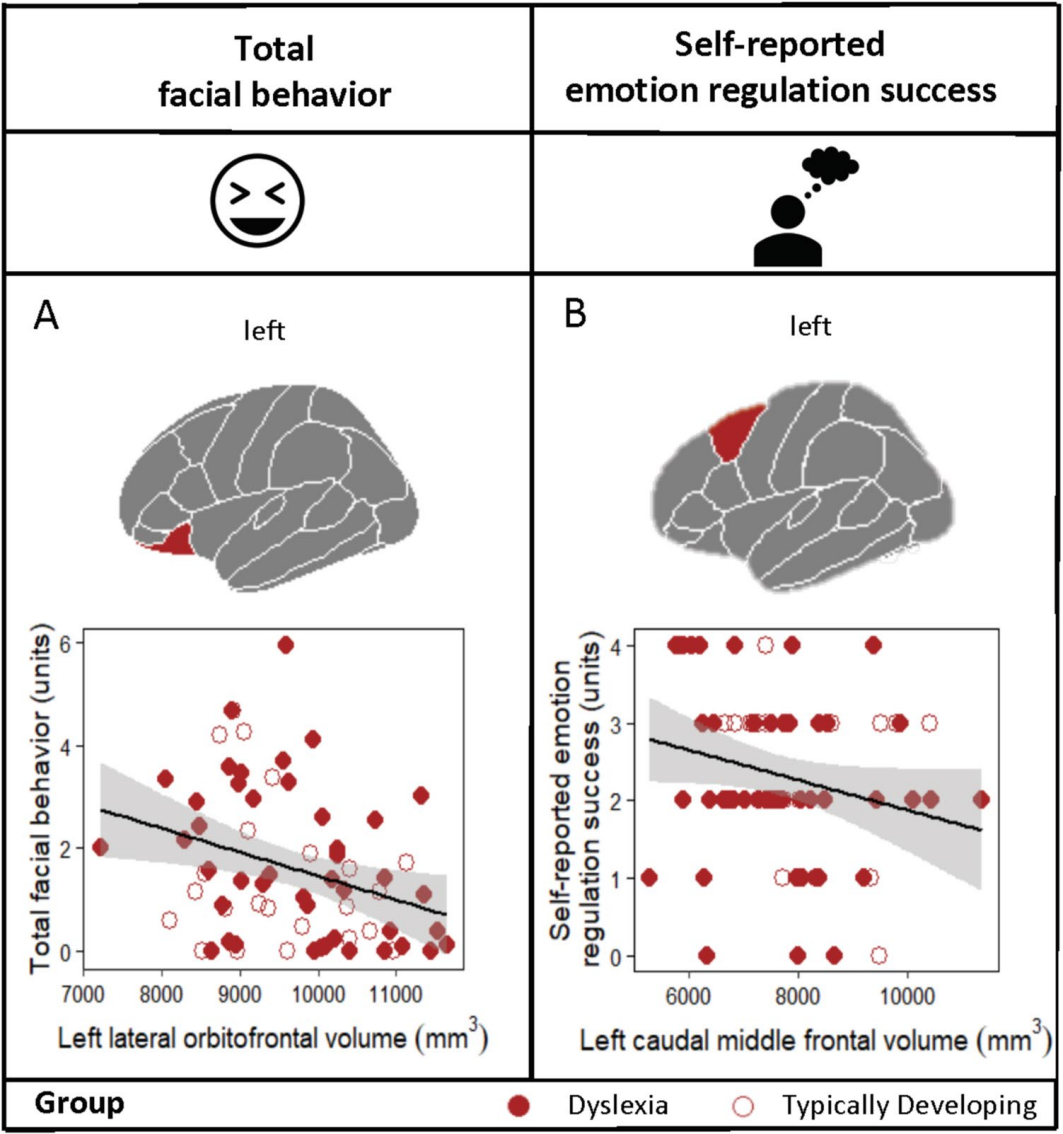
Smaller left PFC gray matter volumes related to worse positive emotion regulation. Lower gray matter volume in (**A**) left lateral orbitofrontal cortex predicted greater total facial behavior, suggesting elevated reactivity during the amusement trial (or worse positive emotion regulation), and (**B**) left dorsolateral prefrontal cortex predicted greater self-reported positive emotion regulation success, suggesting more positive self-evaluation (or greater subjective positive emotion regulation success)

Our findings suggested there was substantial variability in participants’ facial behavior and perceived emotional regulation success during the amusement trial. Although there were no group differences in gray matter volume in any of the ROIs, there was significant variability in these volumes across the children with and without dyslexia (Supplementary Table 6), which we leveraged to examine the neural correlates of positive emotion regulation. Linear regressions across the sample revealed that lower gray matter volume in left lateral OFC predicted greater total facial behavior during the amusement trial (i.e., poorer positive emotion regulation), *Β* = −0.50, *t* = −2.74, *p* =.009. Likewise, lower gray matter volume in the left dorsolateral prefrontal cortex (i.e., caudal middle frontal gyrus) predicted greater self-reported emotion regulation success during the amusement trial (i.e., greater positivity), *Β* = −0.31, *t* = −2.18, *p* =.033 (Fig. 1). In the right hemisphere models, there were no regions that predicted either total facial behavior or self-reported emotion regulation success during the amusement trial. Follow-up analyses using simple linear regressions confirmed that neither the left lateral OFC, *Β* < 0.01, *t* = 1.47, *p* =.147, nor the left dorsolateral PFC, *Β* = −0.02, *t* = −0.13, *p* =.894, predicted emotion regulation during the disgust trial.

### Associations between laboratory-based and parent-reported measures of emotion regulation

**Fig. 2:**
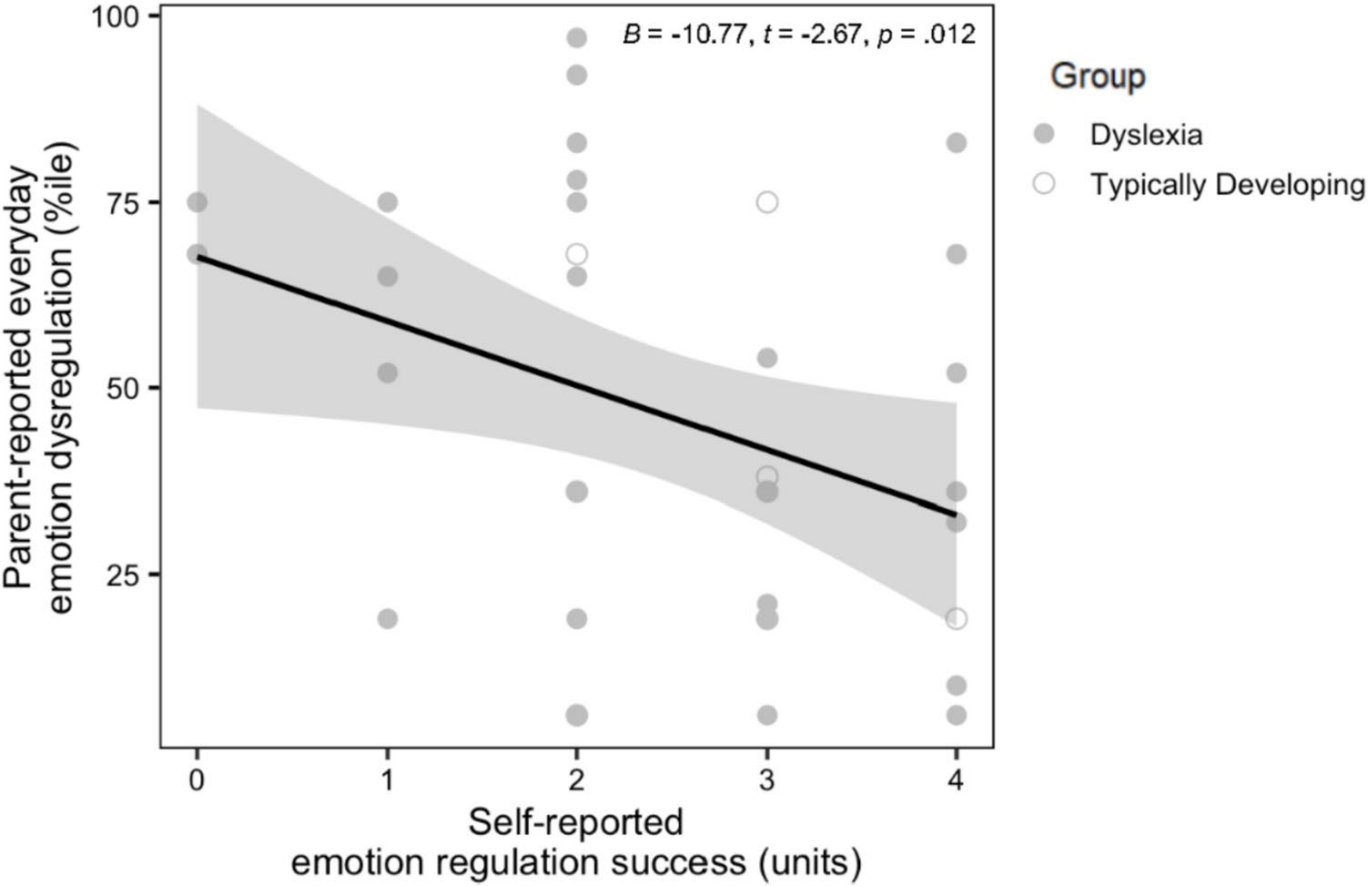
Greater self-reported positive emotion regulation success related to less everyday emotion dysregulation. Across the sample, children with higher self-reported positive emotion regulation success had less parent-reported emotion dysregulation in everyday life (i.e., lower scores on the BASC emotional self-control subscale)

The groups had similar levels of parent-reported emotion regulation in everyday life (Table 2). Greater self-reported emotion regulation success during the amusement trial, *Β* = −10.77, *t* = −2.67, *p* =.012, but not total facial behavior during that trial, *Β* = 0.41, *t* = 0.11, *p* =.916 (Fig. 2), correlated with better real-world emotion regulation. Neither total facial behavior, *Β* = −2.53, *t* = −0.62, *p* =.541, nor self-reported emotion regulation success, *Β* = −0.86, *t* = −0.16, *p* =.872, during the disgust trial related to better real-world emotion regulation, however.

## Discussion

Positive emotions are multicomponential processes that are important for social functioning (Fredrickson, 2013; Fredrickson & Levenson, 1998; Shiota et al., 2011). By leveraging variance in PFC structures across children with and without dyslexia, we found two left-lateral PFC regions related to behavioral and self-reported measures of positive emotion regulation. Lower gray matter volume in the left lateral OFC was associated with greater total facial behavior during the amusement trial while controlling for participants’ emotional reactivity, and lower gray matter volume in the left dorsolateral PFC (i.e., caudal middle frontal gyrus) related to greater self-reported positive emotion regulation success. No right hemisphere regions correlated with either measure of positive emotion regulation. Gray matter volume in these left hemisphere regions was not associated with emotion regulation during the disgust trial. In addition, self-reported positive emotion regulation success related to emotion regulation in everyday life, such that children who reported greater positive emotion regulation success in the laboratory also had better parent-reported emotion regulation in the real world. Because there were no group differences in the laboratory measurements of positive emotion regulation or regional PFC volumes, our findings suggest that even subtle variation in gray matter volume during development may underlie variability in positive emotion regulation, an area of socioemotional functioning that has wide-ranging implications for everyday life.

Our results contribute to an accumulating body of evidence that the left hemisphere, and left-lateral PFC in particular, is crucial for positive emotion regulation. These findings are consistent with our prior studies of older adults that found smaller gray matter volume in left-lateralized emotion regulation systems related to greater smiling behavior in participants while they watched an amusing film clip (Sturm et al., 2015). The current research extends these results to a neurodevelopmental population. Here, smaller volume in left lateral OFC related to greater total facial behavior during the amusement trial, and smaller volume in left dorsolateral PFC related to greater self-reported positive emotion regulation. Our findings suggest that children, like adults, with less robust left-lateral PFC systems may struggle to inhibit positive emotional reactions and tend to have more optimistic assessments of their emotion regulation abilities. The lateral OFC (Beer et al., 2003; Goldin et al., 2008; Ochsner et al., 2012; Öngür & Price, 2000; Phillips et al., 2008) and dorsolateral PFC (Kohn et al., 2014; Phillips et al., 2008) are densely interconnected (Barbas, 2007) and play central roles in cognitive, emotional, and behavioral control. By maintaining goals in working memory, guiding attention, and inhibiting emotion generating systems (Kalisch, 2009; Ochsner & Gross, 2008), these regions contribute to the successful regulation of emotional behavior and experience.

Most prior studies have focused on negative emotion regulation (Carl et al., 2013), but adaptive social functioning also depends on the ability to modulate one’s positive emotions. We found that self-reported emotion regulation success following the positive emotion regulation trial, but not the negative emotion regulation trial, predicted real-world emotion regulation as reported by parents. These results suggest that the children who reported having difficulty regulating their positive emotions in the laboratory tend to have greater everyday struggles with positive emotion regulation. Evidence from adults suggests that being able to suppress positive emotions in certain contexts (e.g., when sharing news of personal success with one’s competitors) is socially adaptive (Erber et al., 1996; Kalokerinos et al., 2014; Stipek, 1998), whereas suppressing positive emotions in contexts where shared experiences are likely (e.g., when receiving a gift from a friend) may be less adaptive (Lyubomirsky et al., 2005). The exuberant child who is so eager to share their experience that they interrupt a parent’s conversation, for example, may have problems with emotion regulation when their approach-related behaviors are thwarted. In childhood, suppression has been associated with both positive (Cole & Jacobs, 2018; Cole et al., 2004; Desatnik et al., 2021; de Veld et al., 2012; Turpyn et al., 2015) and negative (Gardner et al., 2017; Hasking et al., 2017; Kokkinos & Voulgaridou, 2017; Morelen et al., 2016; Lu et al., 2016; Schäfer et al., 2017; Voon et al., 2014) outcomes. More research is needed to understand the contextual factors that may help to explain these conflicting findings.

We also found that children’s self-reported emotion regulation success was a valid assessment of their objective emotion regulation performance given that the children who rated themselves as regulating more successfully during the amusement and disgust trials indeed displayed lower total facial behavior while watching the film clips. Despite these significant associations, the children with smaller left-lateral PFC volumes were more inclined to self-report greater emotion regulation success during the amusement trial, a self-evaluation that seems to be adaptive as it was associated with better parent-reported emotion regulation in everyday life. We speculate that it may have been harder for the children with smaller left-lateral PFC volumes to inhibit their facial behavior during the amusement trial (as they were still highly responsive to the positive stimulus despite trying to inhibit their responses) but easier to view their positive emotion regulation performance in a positive light (regardless of how much facial behavior they displayed). To evaluate their performance during the emotion regulation task, children needed to reflect on and assess how well they had hidden their emotional reaction. Prior studies have found that this type of metacognitive judgment relies on the lateral PFC, although the hemisphere that is involved varies with the type of judgment (Fleming & Dolan, 2012). Developmentally, increasing metacognitive awareness (Zimmer-Gembeck & Skinner, 2011), knowledge of display rules (Gnepp & Hess, 1986; Zeman & Garber, 1996) and self-consciousness (Banerjee, 2002) in middle childhood, abilities that also rely on the PFC (Nelson & Guyer, 2011; Sturm et al., 2006, 2013), coincides with greater emotion regulation (Fuchs & Thelen, 1988). Our results suggest that when self-evaluations pertain to one’s ability to modulate positive emotions, smaller volume in the left PFC may contribute to positive emotion dysregulation and encourage children to see themselves in a positive light, an adaptive ability that gives rise to self-esteem (Beer, 2014; Zell et al., 2020) and engages left PFC circuitry (Parrish et al., 2022; Pauly et al., 2013).

Additional studies are needed to address the present study’s limitations. First, participants were not instructed how to regulate their emotions, but the focus on hiding one’s reaction may have encouraged expressive suppression. Whether children with dyslexia have more pronounced difficulties with language-based emotion regulation strategies, including emotional labeling and cognitive reappraisal, which may be likely given the lower emotion word knowledge we observed here and elsewhere (Palser et al., 2021), will be an important question for future research. Prior studies have shown that naming emotions activates ventrolateral PFC regions, including lateral OFC (Lieberman et al., 2007), and adults who use more refined labels to describe their emotions have greater cortical thickness in lateral areas of the inferior frontal cortex (Lukic et al., 2023). Across development, lateral PFC regions become more connected with the amygdala and more active during reappraisal (McRae et al., 2012; Silvers et al., 2015), and greater cortical thinning in left-lateral PFC during adolescence predicts greater use of cognitive emotion regulation strategies in early adulthood (Vijayakumar et al., 2014). Although these studies suggest that individuals with language disorders may turn to other emotion regulation strategies, additional research is needed to clarify which types of emotion regulation are most impacted. Second, although a surfeit of positive emotions may lead to strengths in dyslexia (Isen et al., 1987; Zhang et al., in press), including entrepreneurship (Fellnhofer, 2023; Logan & Martin, 2012), optimism and resilience (Zhang et al., in press), and creativity (Baas et al., 2008; Majeed et al., 2021), difficulties with negative emotion regulation may also be present and give rise to everyday difficulties. Here, the disgust trial elicited less intense emotional reactions, potentially curtailing our ability to detect associations with PFC volumes or everyday dysregulation. It is likewise possible that participants were able to exert more effort in regulating during the disgust trial, which was presented first, and experienced greater fatigue and therefore less regulatory success during the amusement trial. Interestingly, the children with dyslexia were less optimistic than their peers about their ability to regulate disgust, and showed high levels of concentration on their faces, second only to displays of disgust, suggesting they may have found this trial subjectively difficult (despite not producing more facial behavior than typically developing participants). Future studies that investigate how well children with dyslexia regulate negative emotions, such as fear, anger, and embarrassment, will be important for understanding the full spectrum of emotion regulation abilities in dyslexia and its relation to social functioning. Third, our study was limited in its ability to examine associations between structural anatomy and emotional experience owing to a limited response scale. Developmental research must always balance tailoring experimental paradigms to the abilities of the population while ensuring statistically robust procedures. Replication in older, adolescent participants may allow for greater interrogation of the neural correlates of subjective experience. Finally, the current sample included children with neurodevelopmental disorders living in an urban environment, most of whom were of high socioeconomic status, European descent, and well-resourced. Additional investigations in more diverse samples with additional sociocultural risk factors will be needed to understand whether our findings generalize to broader populations (Deater-Deckard et al., 1998).

The present investigation found evidence for a relationship between positive emotion regulation and left PFC structural anatomy in children with and without dyslexia. By looking across children with and without dyslexia with varying symptoms and brain structure, we were able to identify novel brain-behavior associations (Depue et al., 2010; Fernández-Jaén et al., 2015; Galaburda, 1994; Hoeft et al., 2007; Shultz et al., 2005; Vanderauwera et al., 2017; Vandermosten et al., 2012). Our results suggest that dimensional approaches may be better suited to uncovering the biological mechanisms underlying symptoms in neurodevelopmental conditions rather than focusing on different diagnoses as discrete entities (Astle et al., 2022; England-Mason, 2020). These findings offer new developmental insights into the laterality of emotional functioning and suggest that smaller left hemisphere gray matter volumes may underlie individual differences in positive emotion regulation.

## Supplementary Information

Below is the link to the electronic supplementary material.

Supplementary file1 (DOCX 156 KB)

## Data Availability

Data requests can be submitted thought the UCSF Memory and Aging Center Resource Request form: http://memory.ucsf.edu/resources/data. Academic, not-for-profit investigators with Institutional Review Board approval from the UCSF Human Research Protection Program (HRPP) can request data for research studies. The UCSF HRPP will not review the application until the UCSF Memory and Aging Center Executive Committee has signed off on the proposal and consent form. Data are not publicly available because they contain information that could compromise the privacy of the participants. The experiment was not pre-registered.

## Code availability

Analysis code is available to view at the following Open Science Framework (OSF) location: https://osf.io/reuab/?view_only=43569ca8672b4457b2d61b172110b576
