# Supplementary material for: Role of left lateral prefrontal cortex in positive emotion regulation: Insights from dyslexia
Source: Cogn Affect Behav Neurosci. 2025 Aug 15;25(6):1638–54. doi: 10.3758/s13415-025-01335-8 (PMC12615568; doi:10.3758/s13415-025-01335-8)
Supplement: Supplementary file 1 — Supplementary file1 (DOCX 156 KB) [file 13415_2025_1335_MOESM1_ESM.docx]

**Role of left prefrontal cortex in positive emotion regulation: Insights from dyslexia**

Eleanor R. Palser, Nathaniel A. Morris, Christina R. Veziris, Sarah R. Holley, Amie Wallman-Jones, Ashlin R. K. Roy, Abigail E. Licata, Mieke Voges, Christa Watson Pereira, Maria Luisa Mandelli, Maria Luisa Gorno-Tempini, & Virginia E. Sturm

**Supplementary Materials**

**Supplementary Table 1: Mean and standard deviation (in parentheses) of facial behavior codes by group.**

| **Facial Behavior Code** | **Amusement Trial** | | **Disgust Trial** | |
| --- | --- | --- | --- | --- |
|  | **Dyslexia** | **Typically Developing** | **Dyslexia** | **Typically Developing** |
| Amusement | 11.2 (12.2) | 7.5 (11.5) | 0.8 (2.6) | 0.6 (1.7) |
| Anger | 0.2 (0.6) | 0.1 (0.6) | 0.0 (0.1) | 0.6 (2.3) |
| Concentration | 1.7 (3.6) | 1.3 (2.5) | 3.9 (11.4) | 1.8 (4.5) |
| Contempt | 0.2 (0.7) | 0.0 (0.1) | 0.2 (1.0) | 0.2 (0.4) |
| Disgust | 0 (0) | 0.9 (3.3) | 4.9 (11.6) | 4.1 (10.7) |
| Embarrassment | 1.9 (5.4) | 2.0 (5.9) | 0.4 (2.1) | 0 (0) |
| Fear | 0.0 (0.1) | 0 (0) | 0.3 (1.1) | 0.0 (0.2) |
| Interest | 0.8 (1.7) | 2.0 (5.1) | 2.5 (6.2) | 0.7 (1.5) |
| Sadness | 0.9 (2.7) | 0.4 (1.2) | 2.4 (7.5) | 1.2 (5.1) |

**Supplementary Table 2: Proportion of participants in each group that gave each self-reported emotion regulation response.**

| **Self-Reported Regulation** | **Dyslexia** | **Typically Developing** |
| --- | --- | --- |
| **Amusement Trial** | | |
| **“Very bad”** | 7% | 4% |
| **“Bad”** | 15% | 13% |
| **“OK”** | 38% | 29% |
| **“Good”** | 20% | 46% |
| **“Very good”** | 20% | 8% |
| **NA** | 0% | 0% |
|  | **Disgust Trial** |  |
| **“Very bad”** | 11% | 0% |
| **“Bad”** | 16% | 17% |
| **“OK”** | 53% | 38% |
| **“Good”** | 11% | 29% |
| **“Very good”** | 7% | 17% |
| **NA** | 2% | 0% |

**Supplementary Table 3: Means and standard deviations (in parentheses) by group for self-reported emotional experience during the amusement and disgust regulation trials.**

| **Self-Reported Emotion** | **Dyslexia** | **Typically Developing** |
| --- | --- | --- |
|  | **Amusement Trial** | |
| **Afraid** | 0 (0) | 0 (0) |
| **Amused** | 1.2 (0.9) | 1.3 (0.8) |
| **Angry** | 0.1 (0.4) | 0 (0) |
| **Awe** | 0.3 (0.6) | 0.3 (0.4) |
| **Disgusted** | 0 (0) | 0 (0) |
| **Embarrassed** | 0 (0) | 0 (0) |
| **Excited** | 0.5 (0.8) | 0.5 (0.8) |
| **Love** | 0.1 (0.4) | 0.4 (0.5) |
| **Proud** | 0 (0) | 0 (0) |
| **Sad** | 0 (0.2) | 0 (0) |
| **Surprised** | 0.2 (0.5) | 0.4 (0.6) |
|  | **Disgust Trial** | |
| **Afraid** | 0.0 (0.2) | 0.2 (0.5) |
| **Amused** | 0.1 (0.3) | 0 (0.2) |
| **Angry** | 0.1 (0.4) | 0 (0) |
| **Awe** | 0.1 (0.4) | 0.2 (0.6) |
| **Disgusted** | 1.6 (0.7) | 1.6 (0.7) |
| **Embarrassed** | 0 (0) | 0.1 (0.3) |
| **Excited** | 0 (0) | 0 (0.2) |
| **Love** | 0 (0) | 0 (0) |
| **Proud** | 0 (0) | 0 (0) |
| **Sad** | 0.1 (0.3) | 0 (0) |
| **Surprised** | 0.6 (0.9) | 0.8 (0.7) |

**Supplementary Table 4: Bivariate correlations between self-reported amusement or disgust and other emotion regulation variables.**

| **Amusement Trial** | | |
| --- | --- | --- |
| Self-Reported Amusement | Total Facial Behavior | *r_s_* (66) = 0.10, *p* = .424 |
|  | Amusement Facial Behavior | *r_s_* (66) = 0.17, *p* = .164 |
|  | Self-Reported Emotion Regulation Success | *r_s_* (66) = -0.13, *p* = .274 |
| **Disgust Trial** | | |
| Self-Reported Disgust | Total Facial Behavior | *r_s_* (64) = 0.02, *p* = .847 |
|  | Disgust Facial Behavior | *r_s_* (64) = 0.14, *p* = .254 |
|  | Self-Reported Emotion Regulation Success | *r_s_* (64) = -0.39, *p* = .001* |

**Supplementary Table 5: Bivariate correlations between emotion word knowledge and self-reported emotion regulation variables.**

| **Amusement Trial** | | |
| --- | --- | --- |
| Emotion Word Knowledge | Self-Reported Amusement | *r_s_*(67) = .01, *p* = .958 |
|  | Self-Reported Emotion Regulation Success | *r_s_*(67) = -.02, *p* = .847 |
| **Disgust Trial** | | |
| Emotion Word Knowledge | Self-Reported Disgust | *r_s_*(67) = -.03, *p* = .826 |
|  | Self-Reported Emotion Regulation Success | *r_s_*(67) = .19, *p* = .130 |

**Supplementary Table 6: Means and standard deviations (in parentheses) of volumes (mm^3^) in regions of interest by group. We controlled for TIV, age, sex, and nonverbal reasoning in *F*-tests.**

| **ROI** | **Hemi** | **DYS** | **TD** | ***F*-test** |
| --- | --- | --- | --- | --- |
| Caudal middle frontal | Left | 7714.5 (1309.0) | 7701.7 (1303.5) | *F*(1,61) = 1.34, *p* = .252 |
|  | Right | 7099.1 (1259.8) | 7097.2 (1250.5) | *F*(1,61) = 0.02, *p* = .879 |
| Rostral middle frontal | Left | 20805.0 (2685.6) | 20826.4 (2671.3) | *F*(1,61) = 0.48, *p* = .492 |
|  | Right | 19550.8 (2914.7) | 19565.2 (2895.3) | *F*(1,61) = 1.93, *p* = .170 |
| Caudal anterior cingulate | Left | 2301.9 (567.4) | 2310.8 (567.9) | *F*(1,61) = 0.09, *p* = .769 |
|  | Right | 2259.9 (581.0) | 2256.1 (577.5) | *F*(1,61) = 3.74, *p* = .058 |
| Rostral anterior cingulate | Left | 3482.7 (606.3) | 3479.8 (602.2) | *F*(1,61) = 0.37, *p* = .544 |
|  | Right | 2372.1 (520.7) | 2367.6 (518.1) | *F*(1,61) = 0.25, *p* = .618 |
| Lateral orbitofrontal | Left | 9687.3 (989.9) | 9676.6 (986.4) | *F*(1,61) = 1.17, *p* = .285 |
|  | Right | 9195.7 (1154.1) | 9186.3 (1148.1) | *F*(1,61) = 0.43, *p* = .514 |
| Medial orbitofrontal | Left | 6223.3 (761.8) | 6212.0 (761.9) | *F*(1,61) = 1.17, *p* = .284 |
|  | Right | 6841.8 (765.6) | 6836.4 (761.1) | *F*(1,61) = 0.87, *p* = .354 |
| Pars opercularis | Left | 5806.0 (832.2) | 5799.9 (827.5) | *F*(1,61) = 3.14, *p* = .081 |
|  | Right | 4994.4 (731.3) | 4987.8 (727.9) | *F*(1,61) = 4.77, *p* = .588 |
| Pars triangularis | Left | 4830.4 (635.6) | 4826.5 (631.6) | *F*(1,61) = 1.94, *p* = .169 |
|  | Right | 5349.3 (853.0) | 5348.7 (846.7) | *F*(1,61) = 0.13, *p* = .723 |
| Pars orbitalis | Left | 3144.0 (368.3) | 3138.2 (368.7) | *F*(1,61) = 0.55, *p* = .461 |
|  | Right | 3475.6 (464.0) | 3480.6 (462.4) | *F*(1,61) = 0.00, *p* = .968 |

**Supplementary Figure 1: Associations between total facial behavior and self-reported emotion regulation for the amusement and disgust trials across the sample.**

**
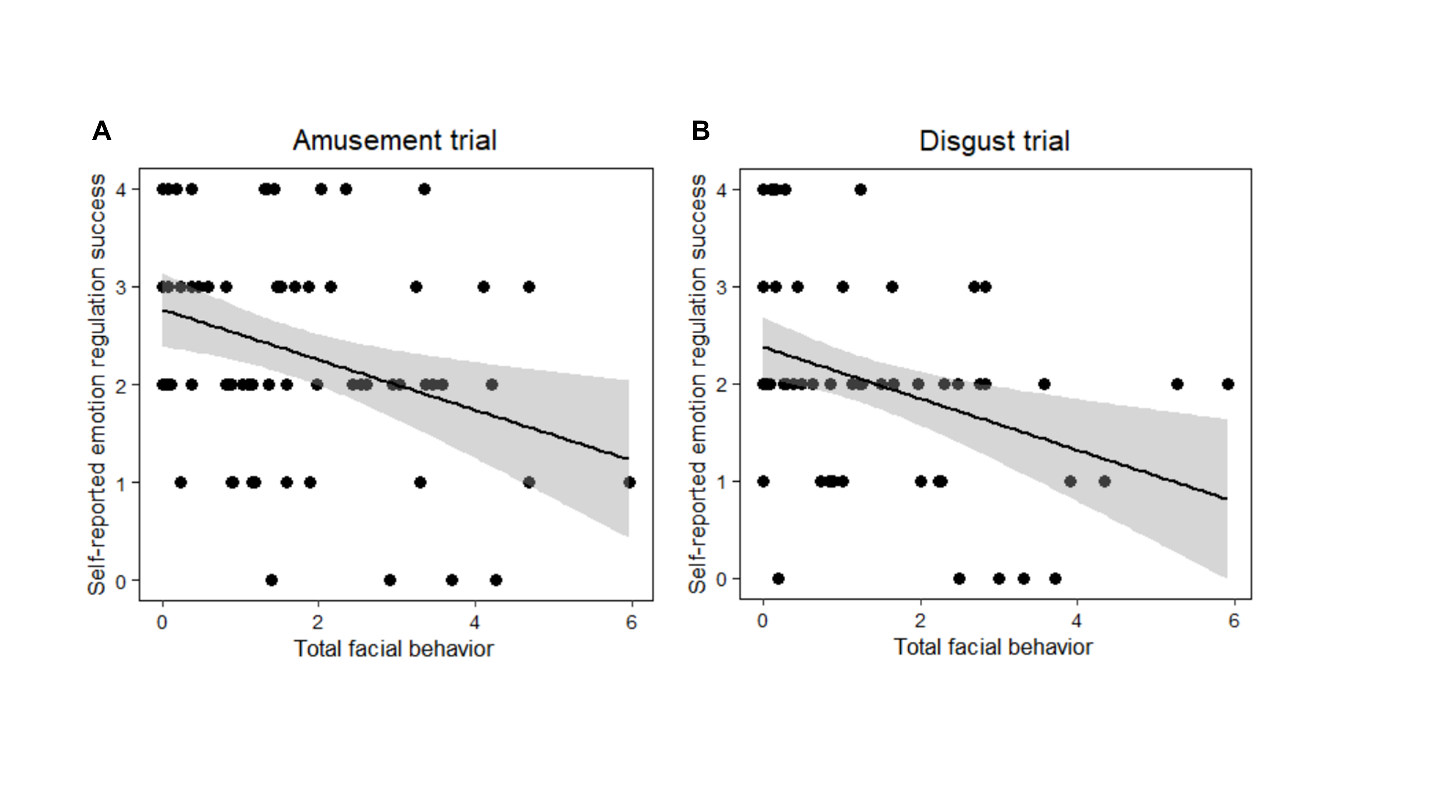
**
